# Evolutionarily recent dual obligatory symbiosis among adelgids indicates a transition between fungus- and insect-associated lifestyles

**DOI:** 10.1038/s41396-021-01056-z

**Published:** 2021-07-22

**Authors:** Gitta Szabó, Frederik Schulz, Alejandro Manzano-Marín, Elena Rebecca Toenshoff, Matthias Horn

**Affiliations:** 1 Department of Microbiology and Ecosystem Science, Centre for Microbiology and Environmental Systems Science, University of Vienna, Vienna, Austria; 2 Present Address: Department of Internal Medicine and Oncology, Semmelweis University, Budapest, Hungary; 3 Present Address: US Department of Energy (DOE) Joint Genome Institute, Berkeley, CA USA; 4 Present Address: Institute of Molecular Biology and Biophysics, ETH Zurich, Zurich, Switzerland

**Keywords:** Environmental microbiology, Microbial ecology

## Abstract

Adelgids (Insecta: Hemiptera: Adelgidae) form a small group of insects but harbor a surprisingly diverse set of bacteriocyte-associated endosymbionts, which suggest multiple replacement and acquisition of symbionts over evolutionary time. Specific pairs of symbionts have been associated with adelgid lineages specialized on different secondary host conifers. Using a metagenomic approach, we investigated the symbiosis of the *Adelges laricis*/*Adelges* *tardus* species complex containing betaproteobacterial (“*Candidatus* Vallotia tarda”) and gammaproteobacterial (“*Candidatus* Profftia tarda”) symbionts. Genomic characteristics and metabolic pathway reconstructions revealed that *Vallotia* and *Profftia* are evolutionary young endosymbionts, which complement each other’s role in essential amino acid production. Phylogenomic analyses and a high level of genomic synteny indicate an origin of the betaproteobacterial symbiont from endosymbionts of *Rhizopus* fungi. This evolutionary transition was accompanied with substantial loss of functions related to transcription regulation, secondary metabolite production, bacterial defense mechanisms, host infection, and manipulation. The transition from fungus to insect endosymbionts extends our current framework about evolutionary trajectories of host-associated microbes.

## Introduction

Most plant sap-feeding insects harbor bacterial endosymbionts, which are of great importance in their host ecology and serve as a model for studying microbe–host relationships and genome evolution of host-restricted bacteria [1–3]. However, how these insect–bacteria partnerships arise is still poorly understood, because the precursor of the endosymbionts can rarely be identified [4–6].

Adelgids (Insecta: Hemiptera: Adelgidae) live on Pinaceae conifers and feed on phloem sap or parenchyma cells [7, 8]. The group has nearly 70 species and is sister to the families of phylloxerans (Phylloxeridae) and aphids (Aphididae) within the suborder Sternorrhyncha. Some adelgid species, such as the balsam woolly adelgid (*Adelges piceae*) and the hemlock woolly adelgid (*Adelges tsugae*), are well-known forest pests and represent severe threats to firs and hemlocks [9].

Adelgids have a complex multigenerational life cycle, which typically involves sexual generations and an alternation between spruce (*Picea*), which is the primary host, and another secondary conifer host (*Abies*, *Pinus*, *Larix*, *Pseudotsuga*, or *Tsuga*). However, other adelgids reproduce asexually on either of the host genera [8].

Similarly to other plant sap-feeding insects, adelgids harbor maternally inherited bacterial symbionts within specialized cells, so-called bacteriocytes, which form a large bacteriome in the abdomen [10–15]. Although the function of these bacterial partners remains largely unexplored, they are expected to provide essential amino acids and B vitamins scarce in the plant sap diet, similarly to obligate endosymbionts of other plant sap-feeding insects [2, 16]. Besides these obligate nutritional endosymbionts, non-essential facultative symbionts might also occur within the bacteriome (or in other tissues), which can provide selective fitness benefits to insects such as protection against parasites and fungal pathogens, increased heat tolerance, or expansion of host plant range [17–19]. Similarly to obligate mutualists, facultative symbionts are usually maternally inherited but can also spread horizontally within and between insect species via mating [20], parasites [21], and food source, such as plant tissues [22].

Interestingly, most adelgid species contain two phylogenetically distinct bacteriocyte-associated symbionts [10, 11, 13–15]. These symbionts belong to at least six different lineages within the Gammaproteobacteria or the Betaproteobacteria. As an exception, in most *A. tsugae* populations a single symbiont resides within the bacteriome, but in each population, there is another symbiont free in the hemocoel [10, 11, 23]. Remarkably, specific pairs of symbionts correspond to distinct lineages of adelgids specialized to one of the five secondary host tree genera [11, 15]. A gammaproteobacterial symbiont lineage involving “*Candidatus* Annandia adelgestsugas” and “*Candidatus* Annandia pinicola” (hereafter *Annandia*), is present in both *A. tsugae* and *Pineus* species and was likely already associated with ancestral adelgids before diversification into the two major adelgid lineages, *Adelges* and *Pineus*, over 87 million years ago [8, 10, 11, 15]. Nevertheless, this putatively ancient symbiont lineage is missing from other adelgids, and the high diversity of symbionts within this small group of insects suggests an evolutionary history involving multiple acquisitions and replacements of bacterial partners [11, 14, 15]. It has been postulated that the frequent turnover of symbionts might be due to fluctuating selective pressure on essential symbiotic functions during evolution coupled to changes in the feeding behavior of adelgids. Adelgids typically feed on nutrient-rich parenchyma versus nutrient-poor phloem on the primary and secondary host trees, respectively, and their host-alternating lifestyles might have emerged repeatedly [11, 23]. The high diversity of symbionts of adelgids is in sharp contrast to the aphid sister group, in which most species have tightly co-evolved with a single obligate symbiont, *Buchnera aphidicola*, for over 180 million years [2].

To date, whole-genome sequences of adelgid endosymbionts are available for only one species: the hemlock woolly adelgid, *A. tsugae* [23]. Metabolic potential and genomic characteristics of *Annandia* resemble those of long-term obligate intracellular symbionts. However, *Annandia* has lost many genes in essential amino acid synthesis. The accompanying, evolutionary more recent *Pseudomonas* symbiont of *A. tsugae*—which is present in the hemocoel—can complement these missing capabilities and thus has a co-obligatory status in the symbiosis [23]. In addition to this obligate dual endosymbiotic system, analysis of a genome fragment of a gammaproteobacterial symbiont (“*Candidatus* Steffania adelgidicola”) of the *Adelges nordmannianae/A. piceae* species complex revealed a metabolically versatile, putatively evolutionarily young endosymbiont in this adelgid lineage [13]. Further genomic data on the symbionts would help to infer the history of association of adelgids with distinct bacterial groups.

Here we investigate the bacterial symbionts of the *Adelges laricis*/*Adelges tardus* species complex using a metagenomic approach and ask what is the function and putative origin of the dual symbiosis in this lineage of adelgids. *A. laricis* and *A. tardus* are morphologically and genetically hardly distinguishable species of adelgids [7, 24]. They contain betaproteobacterial and gammaproteobacterial symbionts, “*Candidatus* Vallotia tarda” and “*Candidatus* Profftia tarda” (hereinafter *Vallotia* and *Profftia*), respectively. Both symbionts are rod shaped and are co-localized inside the same bacteriocytes. *Profftia-*related symbionts have only been found in larch-associated lineages of adelgids, while *Vallotia* symbionts occur in both larch- and Douglas fir-associated lineages of adelgids. Although host–symbiont co-speciation could not be fully resolved with confidence yet, the dual obligatory status of *Profftia* and *Vallotia* in the symbiosis seems to be possible given their common occurrence across different populations and life stages of adelgids [11, 14].

Our results demonstrate that both bacteriocyte-associated symbionts are evolutionary recent partners of adelgids complementing each other’s role in essential amino acid biosynthesis. Notably, phylogenomic analyses revealed a close relationship of *Vallotia* with endosymbionts of *Rhizopus* fungi. Detailed analysis of genomic synteny and gene content indicated an evolutionary transition from fungus to insect symbiosis accompanied by a substantial loss of functions in the insect symbiont especially in transcription regulation, secondary metabolite production, host infection, and manipulation.

## Materials and methods

### Sampling

Spruce (*Picea*) branches with galls of adelgids [7] were collected near Klausen-Leopoldsdorf, Austria (Fig. S1). Galls were stored at −80 °C in the laboratory for subsequent genomic DNA isolation.

### DNA isolation

Before DNA isolation, adelgids were collected from the galls using teasing needles. For high-throughput sequencing, the symbionts were enriched by sequential filtration. The insects were washed twice in buffer A + EDTA solution (35 mM Tris-HCl, 250 mM sucrose, 25 mM KCl, 10 mM MgCl_2_, 250 mM EDTA; pH 7.5) and were subsequently homogenized in fresh solution with a plastic pestle. The suspension was then sequentially filtered through 53 and 30 μm pore size meshes and 5 μm membrane syringe filters. Samples were centrifuged at 7000 rpm for 5 min at 4 °C, and the supernatants were discarded. Pellets were resuspended in buffer A and centrifuged again at 7000 rpm for 5 min at 4 °C. This washing step was repeated once and pellets were next re-suspended in 1×TE buffer (10 mM Tris-HCl, 1 mM EDTA; pH 7.5). High-molecular-weight (HMW) DNA was isolated by an sodium dodecyl sulfate-based DNA extraction method using 1% cetyltrimethylammonium bromide and 1.5% polyvinylpyrrolidone in the extraction buffer [25].

For nanopore sequencing, DNA was isolated from whole insects. Adelgids were rinsed in 1× phosphate-buffered saline three times. DNA was extracted using the Promega Wizard HMW DNA Extraction Kit by homogenizing the adelgids directly in the HMW Lysis Buffer A. The Short Read Eliminator XS Kit from Circulomics was used to remove short DNA fragments.

DNA samples were stored at −20 °C.

### Sequencing and genome assembly

From the symbiont-enriched samples, a paired-end library was sequenced on a HiSeq 2000 sequencer (Illumina). Sequencing reads of 2 × 100 bp were quality filtered and trimmed with PRINSEQ [26] and were assembled with SPAdes v3.1 [27]. Using a subset of 30 million read pairs, a single contig representing the circular *Profftia* chromosome was obtained with 52-fold coverage, while the assembly of the *Vallotia* genome remained fragmented. To improve this assembly, reads were mapped on the *Profftia* genome using the Burrows–Wheeler Alignment (BWA) tool and the BWA-MEM algorithm [28], and matching sequences were removed from further analysis. Contigs >1000 bp obtained in a novel assembly with the remaining reads were further analyzed against a custom protein database containing single-copy markers found in 99% of prokaryote genomes using blastx [29]. Phylogenetic information of the best hits was assessed in Megan v4.70.4 [30]. Ribosomal RNAs (rRNAs) were inferred by RNAmmer [31]. Based on these, seven contigs representing the chromosome and a single contig corresponding to a putative circular plasmid of *Vallotia* have been identified.

In order to close the *Vallotia* genome, we performed nanopore long-read sequencing on an ONT MinION flow cell. Porechop (https://github.com/rrwick/Porechop) and Canu v2.1 [32] were used to remove adapters and for read-error correction, respectively. Nanopore and short reads were mapped against the previously assembled symbiont sequences using Minimap2 v2.17 [33] and Bowtie2 v2.3.5.1 [34], respectively. We pseudo-randomly subsampled 3% of those mapping back to the *Vallotia* contigs, and this read set was used for a targeted reassembly using Unicycler v0.4.9b [35] with *k*-mer sizes of 33, 55, and 77 and in “bold” mode. The novel assembly resulted in two circular molecules corresponding to the chromosome and the previously assembled plasmid of *Vallotia*, with a coverage of 323× and 258×, respectively.

### Genome analysis

The putative origin of replication was identified with GenSkew (http://genskew.csb.univie.ac.at/). We used the ConsPred genome annotation pipeline for gene prediction and annotation [36]. Genome annotations were curated with the help of the UniPro UGENE software [37]. We identified pseudogenes by using the intergenic and hypothetical protein regions as queries in blastx searches against nr and the UniProt Swiss-Prot database with an *e*-value <1e−3 confidence threshold. Pseudogenes were identified as remains of genes, which were either truncated (having a length <80% of the reference) or were interrupted by internal stop codons and/or frameshift mutations. Pseudogene coordinates were set according to the best blast hit in the UniProt Swiss-Prot database, if applicable.

The presence of mobile genetic elements was inferred with blastn and blastx searches against the ISfinder database [38]. Metabolic pathways were explored with the help of the Ecocyc, Biocyc, and Metacyc databases [39] and the Pathway Tools software [40]. Orthologous proteins shared by the relevant genomes or unique to either of the symbionts were identified by using OrthoMCL with a 1e−5 *e*-value threshold [41]. Distribution of the predicted proteins among the main functional categories was explored by using eggNOG-mapper v2 with the DIAMOND sequence comparison option and a 1e−3 *e*-value threshold [42]. Genome alignments were performed by Mauve [43]. Synteny between the chromosomes and plasmids of *Vallotia* and the closely related fungus-associated endosymbiont, *Mycetohabitans rhizoxinica* (accession numbers: FR687359, FR687360) were visualized by Circos based on single-copy shared genes [44]. A close up of collinear regions was created by using the Easyfig tool [45]. The assembled genomes have been submitted to the European Nucleotide Archive under the project accession number PRJEB40648.

### Phylogenetic analyses

A phylogenomic approach was used to infer the phylogenetic positions of the endosymbionts. Protein sequences of closely related species within the *Burkholderiales* and *Enterobacteriales* were collected from the Assembly database of NCBI. Single-copy marker genes were identified by Phyla-AMPHORA [46] using the Brandon Seah (2014) Phylogenomics tools (online: https://github.com/kbseah/phylogenomics-tools). Individual sets of genes were aligned with Muscle 3.8.31 [47]. Poorly aligned positions were removed with Gblocks 0.91b [48] using default parameters apart from the following settings: allowed gap positions with half, the minimum length of a block was 5. Alignments of 108 and 45 proteins were concatenated and used for the calculation of phylogenetic trees for *Vallotia* and *Profftia*, respectively.

For both endosymbionts, we generated maximum likelihood trees with IQ-TREE after selecting the best-fit substitution models with ModelFinder as available at http://iqtree.cibiv.univie.ac.at [49–51]. SH-like approximate likelihood ratio test (SH-aLRT) and ultrafast bootstrap support values, both based on 1000 iterations, were calculated [52]. The best-fit models were LG + F + R5 and LG + R5 for the *Vallotia* and *Profftia* tree, respectively. Besides, Bayesian phylogenetic analyses were performed by MrBayes 3.2.7a [53] with the LG + I + G model and 4 gamma categories on the CIPRES Science Gateway v.3.3. web interface [54]. Two runs, each with 4 chains were performed until convergence diagnostics fell below 0.01. A 50% majority consensus tree was created with a relative burn-in of 25%.

## Results and discussion

### *Profftia* and *Vallotia* are related to free-living bacteria and fungus-associated endosymbionts

Previous 16S rRNA-based phylogenetic analyses suggested an affiliation of *Profftia* with free-living gammaproteobacteria and a close phylogenetic relationship between *Vallotia* and betaproteobacterial endosymbionts of *Rhizopus* fungi [14]. Biased nucleotide composition and accelerated sequence evolution of endosymbiont genomes [2, 3] often result in inconsistent phylogenies and may cause grouping of unrelated taxa [55, 56]. Thus, to further investigate the phylogenetic relationships of the *A. laricis/tardus* symbionts, we used conserved marker genes for maximum likelihood and Bayesian phylogenetic analyses.

Phylogenetic analysis of 45 single-copy proteins demonstrated that *Profftia* opens up a novel insect symbiont lineage most similar to *Hafnia* species and an isolate from the human gastrointestinal tract within the *Hafniaceae*, which has been recently designated as a distinct family within the *Enterobacteriales* [57] (Fig. S2). *Hafnia* strains are frequently identified in the gastrointestinal tract of humans and animals and were also found in insects [58, 59]. The phylogenomic placement of *Profftia* in our analysis is in agreement with previous 16S rRNA-based analyses [14].

**Fig. 1:**
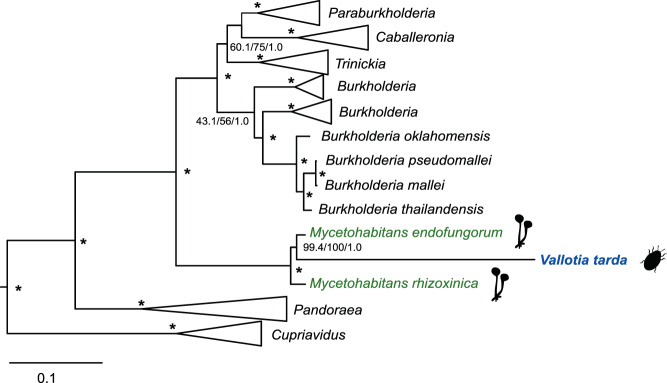
Phylogenomic analysis showing the affiliation of the adelgid endosymbiont “*Candidatus* Vallotia tarda” and its closest relatives, the fungus-associated endosymbionts *M. rhizoxinica* and *M. endofungorum* within the *Burkholderiaceae*. Selected members of *Oxalobacteraceae* (*Janthinobacterium agaricidamnosum* [HG322949], *Collimonas pratensis* [CP013234], and *Herbaspirillum seropedicae* [CP011930]) were used as outgroup. Maximum likelihood and Bayesian analyses were performed based on a concatenated alignment of 108 proteins. Maximum likelihood tree is shown. SH-aLRT support (%) and ultrafast bootstrap support (%) values based on 1000 replicates, and Bayesian posterior probabilities are indicated on the internal nodes. Asterisks stand for a maximal support in each analysis (100%/1).

*Vallotia* formed a monophyletic group with *Mycetohabitans endofungorum* and *M. rhizoxinica*, endosymbionts of *Rhizopus* fungi within the *Burkholderiaceae* [60, 61] with strong support in phylogenetic analyses based on a concatenated set of 108 proteins (Figs. 1 and S3; previous taxonomic assignments of the fungus-associated symbionts were as *Burkholderia*/*Paraburkholderia endofungorum* and *rhizoxinica*, respectively). Interestingly, *Vallotia* and *M. endofungorum* appeared as well-supported sister taxa within this clade. This implies a closer phylogenetic relationship between *Vallotia* and *M. endofungorum* and a common origin of adelgid endosymbionts from within a clade of fungus-associated bacterial symbionts. Lengths of branches leading to the fungus-associated endosymbionts were similar to those of free-living bacteria in the data set; however, *Vallotia* had a remarkably longer branch marking a rapid rate of sequence evolution characteristic of obligate intracellular bacteria [2, 3]. *M. endofungorum* and *M. rhizoxinica* have been identified in the cytosol of the zygomycete *Rhizopus microsporus*, best known as the causative agent of rice seedling blight [61, 62]. The necrotrophic fungus secretes potent toxins, rhizoxin and rhizonin, which are produced by the endosymbionts. The bacterial partners are obligatory for their host as they tightly control its sporulation, while they benefit from host nutrients and spread with the fungal spores [63, 64]. Additionally, related bacterial strains have also been found in association with *Rhizopus* fungi worldwide in a diverse set of environments, including other plant species, soil, food, and even human tissues [65, 66].

Taken together, phylogenomic analyses support that *Profftia* and *Vallotia* open up novel insect symbionts lineages most closely related to free-living bacteria within the *Hafniaceae* and a clade of fungus-associated endosymbionts within the *Burkholderiaceae*, respectively. Given the well-supported phylogenetic positioning of “*Candidatus* Vallotia tarda” nested within a clade formed by *Mycetohabitans* species, we propose the transfer of “*Candidatus* Vallotia tarda” to the *Mycetohabitans* genus, as “*Candidatus* Mycetohabitans vallotii” (a detailed proposal for the re-classification is given in the Supplementary Material).

### *Vallotia* and *Profftia* are evolutionary young symbionts of adelgids

The complete sequence of the *Profftia* chromosome had a length of 1,225,795 bp and a G + C content of 31.9% (Table 1). It encoded for 645 proteins, one copy of each rRNA, 35 transfer RNAs (tRNAs), and 10 non-coding RNAs (ncRNAs). It had tRNAs and amino acid charging potential for all 20 standard amino acids. However, protein-coding sequences (CDSs) made up only 52.4% of the genome, and 21 pseudogenes indicated an ongoing gene inactivation.

**Table 1:** Genomic features of *Profftia* and *Vallotia*.

Organism	Replicon	Genome size (bp)	GC (%)	Coding density (%)	CDS	rRNA gene	tRNA gene	Pseudogene
*Profftia*	Chromosome	1,225,795	31.9	52.4	645	3	35	21
*Vallotia*	Chromosome	1,123,864	42.9	64.9	751	9	41	47
	Plasmid	72,431	36.1	46.2	29	0	0	5

The *Vallotia* chromosome had a length of 1,123,864 bp. It had a G + C content and a coding density of 42.9 and 64.9%, respectively. However, a 72,431-bp-long contig showed a characteristically lower G + C content (36.1%) and contained only 46.2% putative CDSs. This contig had identical repeats at its ends, and genome annotation revealed neighboring genes for a plasmid replication initiation protein, and ParA/ParB partitioning proteins, which function in plasmid and chromosome segregation between daughter cells before cell division [67]. We thus assume that this contig corresponds to a circular plasmid of *Vallotia*. *Vallotia* has three rRNA operons, similarly to its close relative, *M. rhizoxinica* [68]. In total, the *Vallotia* genome encoded 780 proteins (29 on the putative plasmid), 41 tRNAs, and 52 predicted pseudogenes (5 on the putative plasmid).

The host-restricted lifestyle has a profound influence on bacterial genomes. Relaxed purifying selection on many redundant functions and increased genetic drift can lead to the accumulation of slightly deleterious mutations and the proliferation of mobile genetic elements [69–72]. Disruption of DNA repair genes can increase mutation rates, which promote gene inactivation [73]. Non-functional genomic regions get subsequently lost, and ancient obligate endosymbionts typically have tiny (≪0.8 Mb), gene-dense genomes with AT-biased nucleotide composition [2, 74, 75]. Facultative symbionts also possess accelerated rates of sequence evolution but have larger genomes (>2 Mb) with variable coding densities following the age of their host-restricted lifestyle [76]. The number of pseudogenes in *Vallotia* and *Profftia* is higher than in ancient intracellular symbionts, which suggests an intermediate state of genomic reduction [2]. The only moderately reduced size and AT bias together with the low protein-coding density of the *Vallotia* and *Profftia* genomes was most similar to those of evolutionary young co-obligate partners of insects [76], for instance, “*Ca*. Pseudomonas adelgestsugas” in *A. tsugae* [23], *Serratia symbiotica* in *Cinara cedri* [77, 78], and the *Sodalis*-like symbiont of *Philaenus spumarius*, the meadow spittlebug [79].

### The evolutionary link between *Vallotia* and fungus-associated endosymbionts

#### High level of genomic synteny between *Vallotia* and *M. rhizoxinica*

Intracellular symbionts usually show a low level of genomic similarity to related bacteria. Rare examples of newly emerged bacteriocyte-associated symbionts of herbivorous insects pinpoint their source from plant-associated bacteria [4], gut bacteria [5], and other free-living bacteria [6].

Genome alignments showed a low level of collinearity between the genomes of *Profftia* and its closest relatives. Among the relatives of *Vallotia*, a closed genome is available for *M. rhizoxinica* [68]. We therefore mostly focused on this fungus-associated symbiont as a reference for comparison with *Vallotia*.

**Fig. 2:**
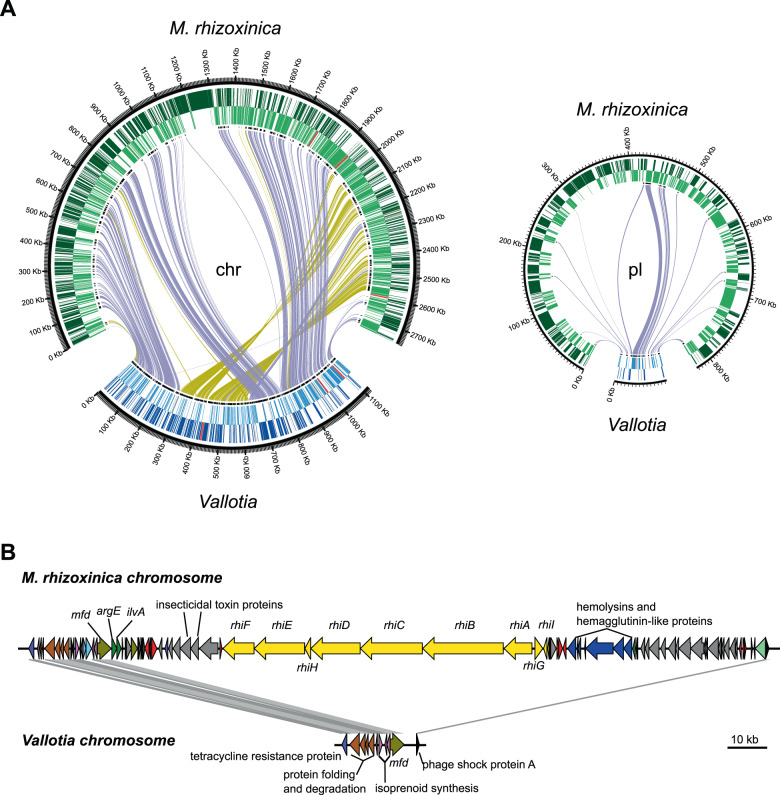
High level of collinearity between the genomes of *Vallotia* and *M. rhizoxinica*. **A** Circos plot showing the synteny between the chromosome and plasmid of *Vallotia* and *M. rhizoxinica*, an endosymbiont of *Rhizopus* fungi. The outermost and the middle rings show genes in forward and reverse strand orientation, respectively. These include rRNA genes in red and tRNA genes in dark orange. The innermost ring indicates single-copy genes shared by *M. rhizoxinica* and *Vallotia* in black. Purple and dark yellow lines connect forward and reverse matches between the genomes, respectively. **B** Close up of the largest deletion on the chromosome of *M. rhizoxinica* and the syntenic region on the *Vallotia* chromosome. Genes are colored according to COG categories. Yellow: secondary metabolite biosynthesis; red: transposase; gray: unknown function; khaki: replication, recombination and repair; pink: lipid transport and metabolism; brown: protein turnover and chaperones; dark green: amino acid transport and metabolism; light green: cell envelope biogenesis; black: transcription. The figure was generated by Easyfig.

The *Vallotia* chromosome showed a surprisingly high level of synteny with the chromosome of *M. rhizoxinica* (Fig. 2A). However, its size was only ~40% of the fungus-associated symbiont chromosome. The putative plasmid of *Vallotia* was perfectly syntenic with the larger of the two plasmids of *M. rhizoxinica* (pBRH01), although the *Vallotia* plasmid was >90% smaller in size (72,431 bp versus 822,304 bp) [68]. Thus, the *Vallotia* plasmid showed a much higher level of reduction than the chromosome, which together with its lower G + C content and gene density suggests differential evolutionary constraints on these replicons.

The conservation of genome structure contrasts with the elevated number of transposases and inactive derivatives making up ~6% of the fungus-associated symbiont genome [68]. Transition to a host-restricted lifestyle is usually followed by a sharp proliferation of mobile genetic elements coupled with many genomic rearrangements [80–82]. However, mobile genetic elements get subsequently purged out of the genomes of strictly vertically transmitted symbionts via a mutational bias toward deletion and because of lack of opportunity for horizontal acquisition of novel genetic elements [71, 74]. Independent origins of the fungus and adelgid symbioses from free-living precursors would have likely resulted in extensive genome rearrangements due to the accumulation of mobile genetic elements, as seen, for instance, between different *S. symbiotica* strains in aphids [81]. In contrast to the fungus-associated symbiont, mobile elements are notably absent from the *Vallotia* genome, suggesting that they might have been lost early after the establishment of the adelgid symbiosis conserving high collinearity between the fungus- and adelgid-associated symbiont genomes. *M. rhizoxinica* is transmitted also horizontally among fungi and might have more exposure to foreign DNA, therefore at least part of the mobile elements could possibly be inserted into its genome after the host switch of the *Vallotia* precursor [61, 62].

The observed high level of genome synteny between *Vallotia* and *M. rhizoxinica* genomes is consistent with the phylogenetic position of *Vallotia* interleaved within the clade of *Rhizopus* endosymbionts. This points toward a direct evolutionary link between these symbioses and a symbiont transition between the fungus and insect hosts.

#### Shrinkage of the insect symbiont genome

Deletion of large genomic fragments—spanning many functionally unrelated genes—represents an important driving force of genome erosion especially at early stages of symbioses when selection on many functions is weak [3, 83]. Besides, gene loss also occurs individually and is ongoing, albeit at a much lower rate, even in ancient symbionts [75, 84, 85]. Both small and large deletions could be seen when comparing the *Vallotia* and *M. rhizoxinica* genomes. Several small deletions as small as one gene were observed sparsely in the entire length of the *Vallotia* genome within otherwise collinear regions. The largest genomic region missing from *Vallotia* encompassed 165 kbp on the *M. rhizoxinica* chromosome (Fig. 2B). The corresponding intergenic spacer was only 3843-bp long on the *Vallotia* genome between a phage shock protein and the Mfd transcription-repair-coupling factor, present both in *Vallotia* and *M. rhizoxinica*. Interestingly, this large genomic fragment included the large rhizoxin biosynthesis gene cluster (*rhiIGBCDHEF*), which is responsible for the production of rhizoxin, a potent antimitotic macrolide serving as a virulence factor for *R. microsporus*, the host of *M. rhizoxinica* [86]. A homologous gene cluster was also found in *Pseudomonas fluorescens*, and it has been suggested that it has been horizontally acquired by *M. rhizoxinica* [68, 86]. The *rhi* cluster is also present in *M. endofungorum*, therefore it was most likely already present in the genome of the common ancestor of the fungus- and adelgid-associated symbionts and got subsequently lost in *Vallotia*. Rhizoxin blocks microtubule formation in various types of eukaryotic cells [86, 87], thus the loss of this gene cluster in ancestral *Vallotia* could have contributed to the establishment of the adelgid symbiosis. However, this large deleted genomic region also contained several transposases and many other genes, such as *argE* and *ilvA*, coding for the final enzymes for ornithine and 2-oxobutanoate productions, which were located adjacent to each other at the beginning of this fragment. The largest deletion between the plasmids encompassed nearly 137 kbp of the megaplasmid of *M. rhizoxinica* and involved several non-ribosomal peptide synthetases (NRPS), insecticidal toxin complex (Tc) proteins, and a high number of transposases among others. *M. rhizoxinica* harbors 15 NRPS gene clusters [68] in total, all of which are absent in *Vallotia*. NRPSs are large multienzyme machineries that assemble various peptides, which might function as antibiotics, signal molecules, or virulence factors [88]. Insecticidal toxin complexes are bacterial protein toxins, which exhibit powerful insecticidal activity [89]. Two of such proteins are also present in the large deleted chromosomal region in close proximity to the rhizoxin biosynthesis gene cluster (Fig. 2B); however, their role in *M. rhizoxinica* remains elusive.

#### The *Vallotia* genome encodes a subset of functions of the fungus-associated endosymbionts

The number of protein-coding genes of *Vallotia* is less than one-third of those of *M. rhizoxinica* and *M. endofungorum*, although metabolic functions are already reduced in the fungus-associated endosymbionts compared to free-living *Burkholderia* species [68] (Figs. S4 and S5). When compared to the two genomes of the fungus-associated endosymbionts, only 53 proteins were specific to *Vallotia* (Fig. S6). All of these were short (on average 68 amino acid long) hypothetical proteins and most of them showed no significant similarity to other proteins in public databases. Whether these *Vallotia*-specific hypothetical proteins might be over-annotated/non-functional open reading frames or orphan genes with a yet unknown function [90, 91] needs further investigation. Four genes were present in *Vallotia* and *M. rhizoxinica* but were missing in *M. endofungorum*. These encoded for BioA and BioD in biotin biosynthesis, NagZ in cell wall recycling, and an MFS transporter. Fifteen genes, including, for instance, the MreB rod-shape-determining protein, glycosyltransferase and hit family proteins, genes in lipopolysaccharide, lipoate synthesis, and the oxidative pentose phosphate pathway, were shared between *Vallotia* and *M. endofungorum* only. The rest of the *Vallotia* genes, coding for 91% of all of its proteins, were shared among the fungus- and insect-associated endosymbionts.

Comparing the genes present in both endosymbionts to those shared only by the fungus-associated endosymbionts (Fig. S7), we can infer selective functions maintained or lost during transition to insect endosymbiosis. Translation-related functions have been retained in the greatest measure in the group shared by all endosymbionts. Functions, where higher proportion of genes were specific to the fungus endosymbioses, were related to transcription, inorganic ion transport and metabolism, secondary metabolite biosynthesis, signal transduction, intracellular trafficking, secretion, vesicular transport, and defense mechanisms. Most of the proteins specific to either of the fungus-associated symbionts were homologous to transposases and integrases, transcriptional regulators, or had an unknown function.

Fungus-associated endosymbionts encode a high number of transcriptional regulators (~5% of all genes in *M. rhizoxinica*) [68], but *Vallotia* has retained only a handful of such genes, which is a feature similar to other insect symbionts and might facilitate the overproduction of essential amino acids [75, 92].

*M. rhizoxinica* is resistant against various β-lactams and has an arsenal of efflux pumps that might provide defense against antibacterial fungal molecules, the latter might also excrete virulence factors to the fungus cytosol (type I secretion) [68]. Besides, *M. rhizoxinica* encodes several genes for pilus formation; adhesion proteins; and type II, type III, and type IV secretion systems, which likely play a central role in host infection and manipulation in the bacteria–fungus symbiosis [68, 93, 94]. However, all of the corresponding genes are missing in *Vallotia*. Thus, neither of these mechanisms likely play a role in the adelgid symbiosis. Indeed, we could not even detect remnants of these genes in the *Vallotia* genome, except for a type II secretion system protein as a pseudogene. Loss of these functions is consistent with a strictly vertical transmission of *Vallotia* between host generations. Transovarial transmission likely does not require active infection mechanisms, and the endosymbionts rather move between the insect cells in a passive manner via an endocytic/exocytic vesicular route [12, 95]. In contrast, *M. rhizoxinca* is also able to spread horizontally among fungi and re-infect cured *Rhizopus* strains under laboratory conditions [61, 62].

### Differential reduction of metabolic pathways in *Vallotia* and *Profftia*

Although compared to their closest free-living relatives both *Vallotia* and *Profftia* have lost many genes in all functional categories, both retained the highest number of genes in translation-related functions (Fig. S4). Besides, functions related to cell division, nucleotide and coenzyme transport and metabolism, DNA replication and repair, posttranslational modification, and cell envelope biogenesis are reduced to a lesser extent in both endosymbionts. As a consequence, most of the genes of *Vallotia* and *Profftia* are devoted to translation and cell envelope biogenesis, which make up higher proportions of their genomes than in related bacteria (Fig. S5). Retention of a minimal set of genes involved in central cellular functions such as translation, transcription, and replication is a typical feature of reduced genomes, even extremely tiny ones of long-term symbionts [75]. However, ancient intracellular symbionts usually miss a substantial number of genes for the production of the cell envelope and might rely on host-derived membrane compounds [96–98].

Based on pathway reconstructions, both *Vallotia* (Fig. S8) and *Profftia* (Fig. S9) have a complete gene set for peptidoglycan, fatty acid, and phospholipid biosynthesis and retained most of the genes for the production of lipid A, LPS core, and the Lpt LPS transport machinery. Besides, we found a partial set of genes for O antigen biosynthesis in the *Vallotia* genome. Regarding the membrane protein transport and assembly, both adelgid endosymbionts have the necessary genes for Sec and signal recognition particle translocation and the BAM outer membrane protein assembly complex. *Profftia* also has a complete Lol lipoprotein trafficking machinery (*lolABCDE*), which can deliver newly matured lipoproteins from the inner membrane to the outer membrane [99]. In addition, *Profftia* has a near-complete gene set for the Tol-Pal system; however, *tolA* has been pseudogenized suggesting an ongoing reduction of this complex. Further, both adelgid endosymbionts have retained *mrdAB* and *mreBCD* having a role in the maintenance of cell wall integrity and morphology [100, 101]. The observed well-preserved cellular functions for cell envelope biogenesis and integrity are consistent with the rod-shaped cell morphology of *Profftia* and *Vallotia* [14], contrasting the spherical/pleomorphic cell shape of ancient endosymbionts, such as *Annandia* in *A. tsugae* and *Pineus* species [10, 11, 15].

Regarding the central metabolism, *Vallotia* lacks 6-phosphofructokinase but has a complete gene set for gluconeogenesis and the tricarboxylic acid (TCA) cycle. TCA cycle genes are typically lost in long-term symbionts but are present in facultative and evolutionarily recent obligate endosymbionts [79, 82, 102]. Interestingly, *Vallotia* does not have a recognized sugar transporter. Similarly to *M. rhizoxinica* [68], a glycerol kinase gene next to a putative glycerol uptake facilitator protein is present on its plasmid. However, the latter gene has a frameshift mutation and a premature stop codon in the first 40% of the sequence and whether it can still produce a functional protein remains unknown.

*Profftia* can convert acetyl-CoA to acetate for energy but lacks TCA cycle genes, a feature characteristic to more reduced genomes, such as, for instance, *Annandia* in *A. tsugae* [23]. *Profftia* has import systems for a variety of organic compounds, such as murein tripeptides, phospholipids, thiamine, spermidine and putrescine, 3-phenylpropionate, and a complete phosphotransferase system for the uptake of sugars.

NADH dehydrogenase, ATP synthase, and cytochrome oxidases (*bo*/*bd*-1) are encoded on both adelgid symbiont genomes. However, *Vallotia* is not able to produce ubiquinone and six pseudogenes in its genome indicate a recent inactivation of this pathway (Fig. S10).

*Profftia* retained more functions in inorganic ion transport and metabolism, while *Vallotia* had a characteristically higher number of genes related to amino acid biosynthesis (see its function below) and nucleotide transport and metabolism (Fig. S4). For instance, *Profftia* can take up sulfate and use it for assimilatory sulfate reduction and cysteine production, and it has also retained many genes for heme biosynthesis (Fig. S9). However, it cannot produce inosine-5-phosphate and uridine 5’-monophosphate precursors for the de novo synthesis of purine and pyrimidine nucleotides and thus would need to import these compounds.

Notably, although core genes in DNA replication and repair [70] are well preserved, multiple pseudogenes may indicate an ongoing erosion of DNA repair functions in the genomes. These include genes for the UvrABC nucleotide excision repair complex in both adelgid symbionts, helicases (*recG*, *recQ*), mismatch repair genes (*mutL*, *mutS*; the MutHLS complex is also missing in *Profftia*), and *alkA* encoding a DNA glycosylase in *Vallotia*.

Taken together, their moderately reduced, gene-sparse genomes but still versatile metabolic capabilities support that *Vallotia* and *Profftia* are evolutionarily recently acquired endosymbionts. This is following their occurrence in lineages of adelgids, which likely diversified relatively recently, ~60 and ~47 million years ago, respectively, from the remaining clades of Adelgidae [8].

### *Vallotia* and *Profftia* are both obligatory nutritional symbionts

#### Complementary functions in essential amino acid provision

**Fig. 3:**
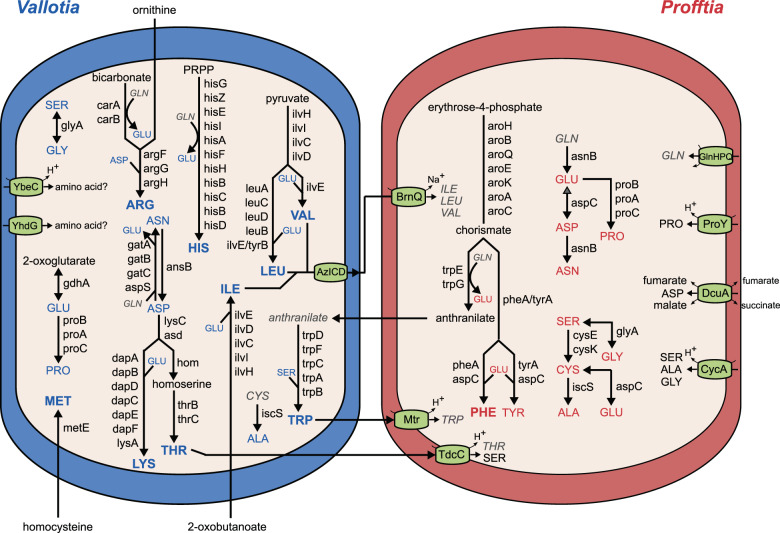
Division of labor in amino acid biosynthesis and transport between *Vallotia* and *Profftia* showing co-obligatory status of endosymbionts of *A. laricis/tardus*. Amino acids produced by *Vallotia* and *Profftia* are shown in blue and red, respectively. Bolded texts indicate essential amino acids. The insect host likely supplies ornithine, homocysteine, 2-oxobutanoate, and glutamine. Other compounds that cannot be synthesized by the symbionts are shown in gray italics.

*Vallotia* and *Profftia* complement each other’s role in the essential amino acid synthesis, thus have a co-obligatory status in the *A. laricis*/*A. tardus* symbiosis (Fig. 3). Although *Vallotia* likely generates most essential amino acids, solely *Profftia* can produce chorismate, a key precursor for the synthesis of phenylalanine and tryptophan. *Profftia* is likely responsible for the complete biosynthesis of phenylalanine as it has a full set of genes for this pathway. It can also convert chorismate to anthranilate; however, further genes for tryptophan biosynthesis are only present in the *Vallotia* genome. Thus, *Vallotia* likely takes up anthranilate for tryptophan biosynthesis. Anthranilate synthase (*trpEG*), is subject to negative feedback regulation by tryptophan [103], thus partition of this rate-limiting step between the co-symbionts can enhance overproduction of the amino acid and might stabilize dual symbiotic partnerships at an early stage of coexistence. The production of tryptophan is partitioned between *Vallotia* and *Profftia* similarly as seen in other insect symbioses [77, 78, 104], and it is also shared but is more redundant between the *Annandia* and *Pseudomonas* symbionts of *A. tsugae* [23]. The *Vallotia*–*Profftia* system generally shows a lower level of functional overlap between the symbionts and is more unbalanced than the *Annandia*–*Pseudomonas* association. In the latter, redundant genes are present also in the synthesis of phenylalanine, threonine, lysine, and arginine, and *Annandia* can produce seven and the *Pseudomonas* partner five essential amino acids with the contribution of host genes [23].

The *Vallotia* genome encodes for all the enzymes for the synthesis of five essential amino acids (histidine, leucine, valine, lysine, threonine). *ArgG* and *tyrB* among the essential amino acid synthesis-related genes are only present on the plasmid of *Vallotia*, which might be a reason that the plasmid is still part of its genome. However, neither of the endosymbionts can produce ornithine, 2-oxobutanoate, and homocysteine de novo, which are key for the biosynthesis of arginine, isoleucine, and methionine, respectively. The corresponding functions are also missing from the *Annandia*–*Pseudomonas* system [23]. These compounds are thus likely supplied by the insect host, as seen for instance in aphids, mealybugs, and psyllids, where the respective genes are present in the insect genomes and are typically overexpressed within the bacteriome [97, 105, 106]. The *metC* and *argA* genes are still present as pseudogenes in *Vallotia* suggesting a recent loss of these functions in methionine and arginine biosynthesis, respectively.

In most plant sap-feeding insects harboring a dual symbiotic system, typically the more ancient symbiont provides most of the essential amino acids [77, 107]. Given its prominent role in nutrient provision and its presence in both larch- and Douglas fir-associated adelgids, *Vallotia* might be the older symbiont. Loss of functions in chorismate and anthranilate biosynthesis might have led to the fixation of *Profftia* in the system.

*Vallotia* and *Profftia* have more redundant functions in non-essential amino acid production (Fig. 3). Only *Profftia* can produce cysteine and tyrosine, while none of the symbionts can build up glutamine, thus this latter amino acid is likely supplied by the insect bacteriocytes.

The presence of relevant transporters can complement missing functions in amino acid synthesis (Fig. 3). For instance, *Profftia* has a high-affinity glutamine ABC transporter and three symporters (BrnQ, Mtr, TdcC), which can import five among the essential amino acids that can be produced by *Vallotia*. *Vallotia* might excrete isoleucine, valine, and leucine via AzICD, a putative branched-chain amino acid efflux pump [108], and these amino acids could be taken up by *Profftia* via BrnQ and would be readily available also for the insect host.

#### B vitamin provision by *Vallotia*

Regarding the B vitamin synthesis, *Vallotia* is likely able to produce thiamine (B_1_), riboflavin (B_2_), pantothenate (B_5_), pyridoxine (B_6_), biotin (B_7_), and folic acid (B_9_) (Fig. S11). Although *Vallotia* misses some genes of the canonical pathways, alternative enzymes and host-derived compounds might bypass these reactions, as detailed in the Supplementary Material. *Profftia* has only a few genes related to B vitamin biosynthesis. Three pseudogenes (*ribAEC*) in the riboflavin synthesis pathway indicate that these functions might have been lost recently in this symbiont (Fig. S11).

## Conclusions

Our results demonstrated that both endosymbionts of *A. laricis*/*A*. *tardus* are evolutionary recent bacterial partners of adelgids with moderately reduced genomes. *Profftia* and *Vallotia* open up novel insect symbiont lineages within the *Hafniaceae* and *Burkholderiaceae*, respectively. Given its phylogenetic position within a clade of *Mycetohabitans* species, we propose the reclassification of the betaproteobacterial symbiont as “*Candidatus* Mycetohabitans vallotii”.

A direct evolutionary link between *Vallotia* and endosymbionts of *Rhizopus* fungi was supported by a high level of genomic synteny. The phylogenetic position of *Vallotia* interleaved within the clade of *Rhizopus* endosymbionts and the lack of functions specific to the adelgid symbiont point toward a transition of endosymbiosis from fungi to insect hosts. The evolutionary origin of insect symbionts from fungus-associated endosymbionts is, according to our knowledge, unprecedented. *Rhizopus* endosymbionts are equipped with many functions for infection and overcoming host defense. Chitinase, chitosanase, and a putative chitin-binding protein have also been found among the putatively *Sec* exported proteins of *M. rhizoxinica* [68], which besides the infection of fungi could have had a role in the transmission into an insect. Their host, *R. microsporus*, is a plant pathogen fungus with a broad environmental distribution. Thus, a potential route for acquisition of the symbiont by insects could have been via plant tissues, the food source of adelgids, similar to plant-mediated symbiont transmission observed for intracellular insect symbionts [22].

*Vallotia* has a pivotal role in essential nutrient provision, but both endosymbionts are obligatory to their insect host. It has been suggested that repeated replacements of symbionts among adelgids might be a consequence of periods with relaxed selection on symbiont functions due to different feeding behavior of adelgids on primary and secondary host trees and multiple origins of their host-alternating lifestyles [11]. *Annandia*, the ancient symbiont of adelgids, has lost many functions in essential amino acid biosynthesis, which could support this hypothesis [23], although the *Vallotia*–*Profftia* system does not show this pattern.

Taken together, our comprehensive genomic analysis of co-obligate endosymbionts of adelgids revealed a novel path for the evolution of bacteria–insect symbioses from a clade of fungus-associated ancestors.

## Supplementary information

Supplementary Information
