# Supplementary material for: Evolutionarily recent dual obligatory symbiosis among adelgids indicates a transition between fungus- and insect-associated lifestyles
Source: ISME J. 2021 Jul 22;16(1):247–56. doi: 10.1038/s41396-021-01056-z (PMC8692619; doi:10.1038/s41396-021-01056-z)
Supplement: Supplementary file 1 — Supplementary Information [file 41396_2021_1056_MOESM1_ESM.pdf]

Evolutionarily recent dual obligatory symbiosis among adelgids indicates a transition between fungus- and insect-associated lifestyles

Gitta Szabó, Frederik Schulz, Alejandro Manzano-Marín,
Elena Rebecca Toenshoff, Matthias Horn

Supplementary Information

'*Candidatus* Mycetohabitans vallotii' ***sp. nov.***

Based on the well supported phylogenetic positioning of *'Candidatus* Vallotia tarda' within the clade formed by both currently recognized *Mycetohabitans* species, we propose the transfer of *'Candidatus* Vallotia tarda' (NCBI taxonomy ID 1177213) to the *Mycetohabitans* genus. To keep the naming consistent, we propose the specific name *'Candidatus* Mycetohabitans vallotii' (va.lo.tii) in honor of the researcher Vallot, who described *A. laricis* in 1836. *'Candidatus* Mycetohabitans vallotii' strains have a rod-shaped cell and co-inhabit the cytoplasm of bacteriocytes of *A. laricis/tardus* along with *'Candidatus* Profftia tarda' [1]. We propose the old species-specific name be used as a strain name, as '*Candidatus* Mycetohabitans vallotii' strain tarda. Given their monophyletic origin, the transfer of other species of the '*Candidatus* Vallotia' genus to the *Mycetohabitans* genus is reasonable [1, 2], however as multilocus sequence data are not available for those endosymbionts yet, we leave their species level re-designation open for future studies.

B vitamin synthesis by *Vallotia*

Vallotia likely produces six B vitamins (Figures S8, S11). For the synthesis of riboflavin, neither *yigB* nor *ybjI* [3] were found, however, the promiscuity of phosphatases has been documented [4]. Thus another phosphatase of *Vallotia*, among those belonging to the Haloacid dehalogenase-like hydrolase superfamily, like YigB, might perform this reaction. As is the case for other endosymbionts, *panD* is missing for the *de novo* synthesis of pantothenate, thus this would occur from L-valine and β-alanine in *Vallotia*. Regarding pyridoxine, *Vallotia* lacks *pdxB* and *serC*, but alternatives such as the 'serendipitous pathways', *thiG* and an unspecific transaminase might bypass these steps [5, 6]. *BioH* and *bioF* in biotin synthesis are missing from both symbionts, nonetheless, these are also notably absent in several symbiotic systems of aphids [7, 8]. *Vallotia* might still produce biotin, if either 8-amino-7-oxononanoate (KAPA) is imported or if these steps are taken over by the host. Finally, given their lack of *nadB* and *nadC* genes, both endosymbionts could synthesize NAD+ and NADP+ from the import of nicotinate.

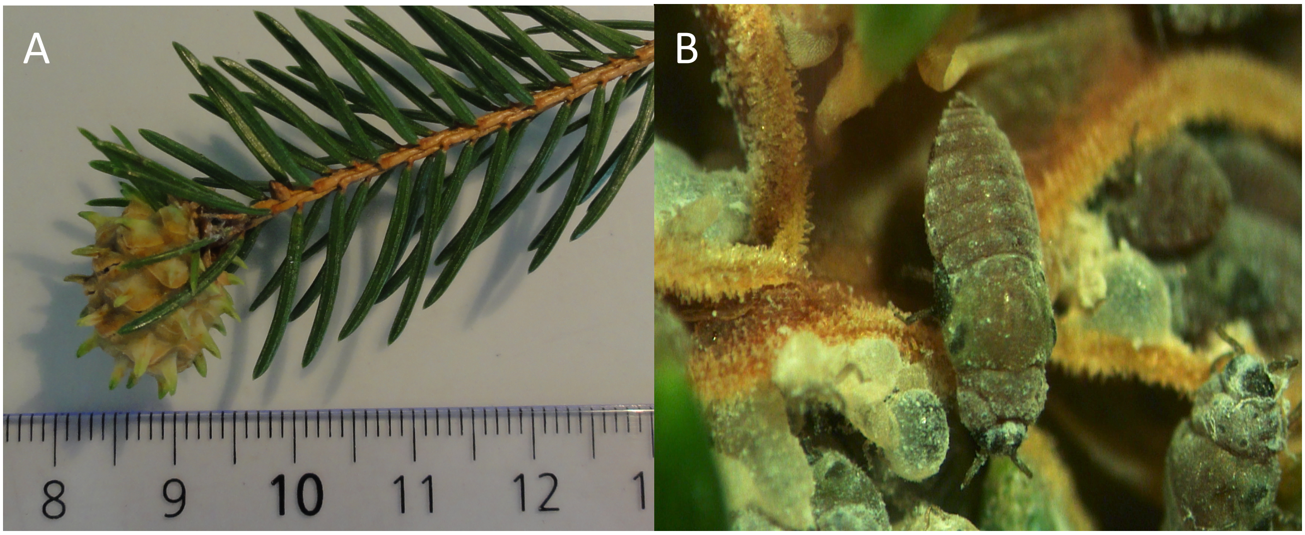

Figure S1. (A) A gall of *Adelges laricis/tardus* collected with a spruce branch. **(B)** Adelgids in an opened gall.

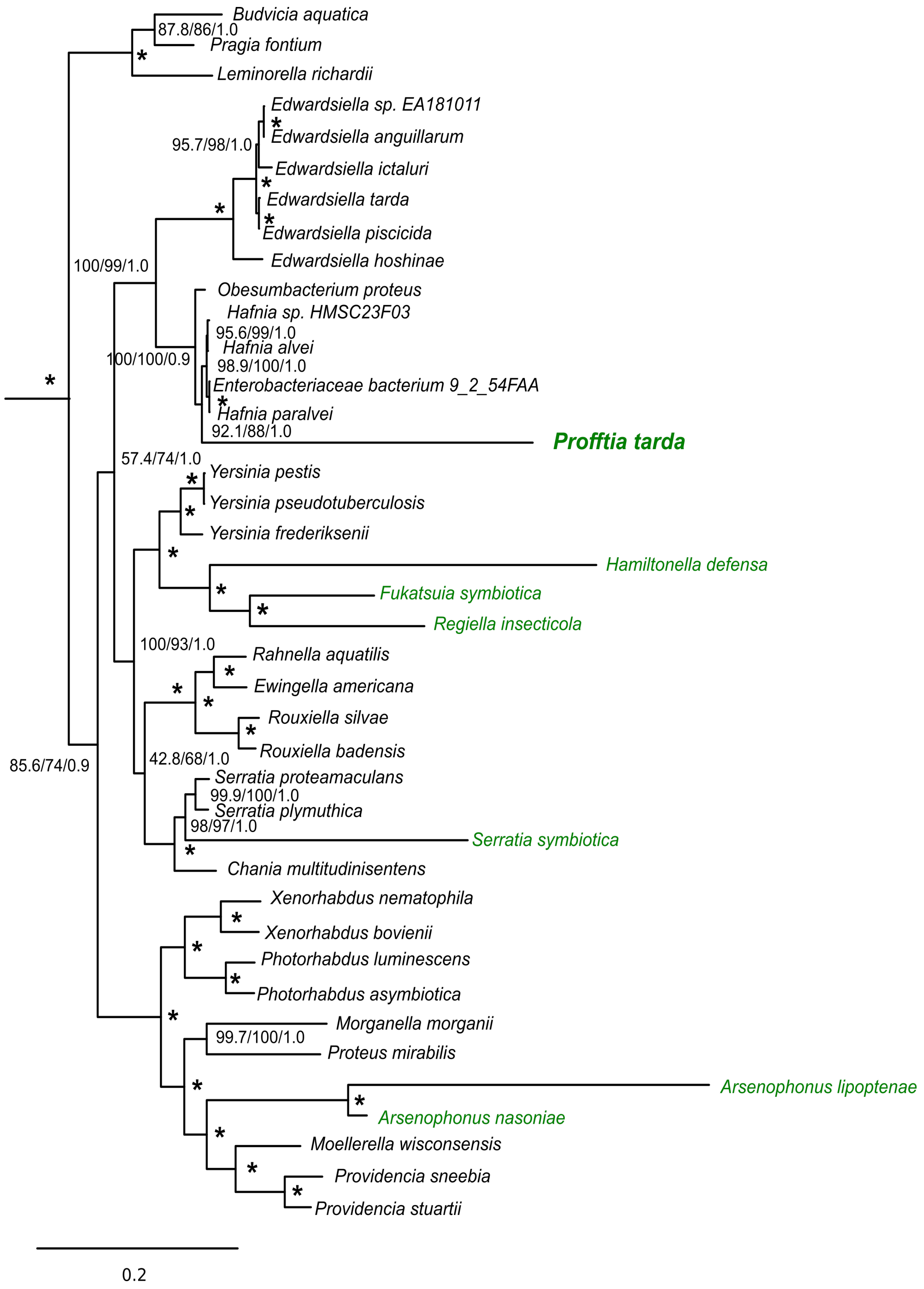

Figure S2. Phylogenomic analysis showing the affiliation of '*Candidatus* Profftia tarda' within the Enterobacteriales. Insect symbionts are highlighted in green. *Xanthomonas campestris* [AE008922], *Stenotrophomonas maltophilia* [AM743169] and *Pseudomonas aeruginosa* [AE004091]) were used as outgroups. Maximum likelihood (IQTREE) and Bayesian trees (MrBayes) were based on a concatenated set of 45 proteins. Maximum likelihood tree is shown. SH-aLRT support (%) and ultrafast bootstrap support (%) values based on 1000 replicates, and Bayesian posterior probabilities are indicated on the internal nodes. Asterisks stand for a maximal support in each analysis (100% / 1). The branch length leading to *Profftia* indicated accelerated evolutionary rates and was similar to those of other obligate and facultative insect symbionts included in the analysis.

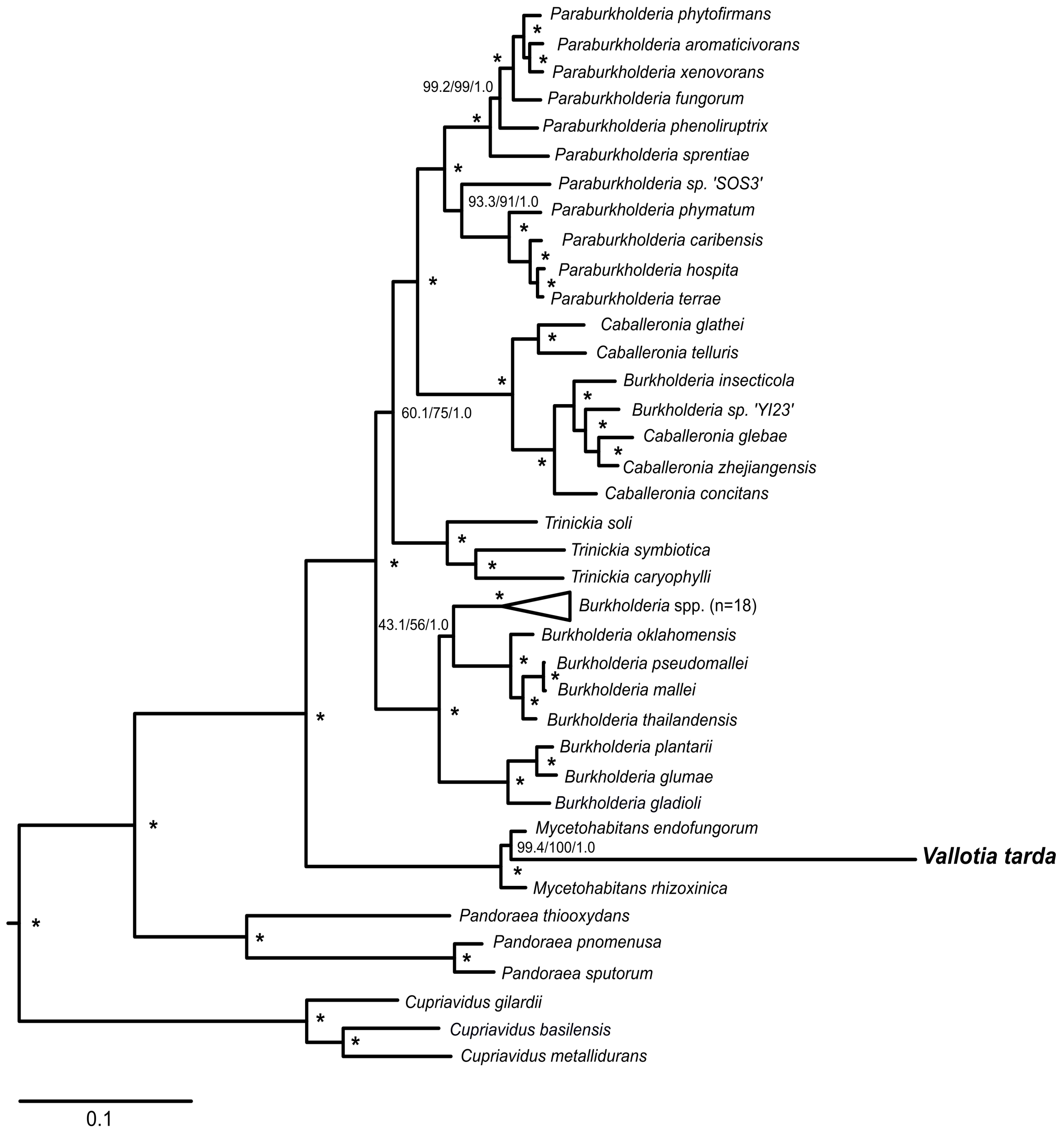

Figure S3. Phylogenomic analysis showing the affiliation of '*Candidatus* Vallotia tarda' within the *Burkholderiaceae*. Selected members of *Oxalobacteraceae* (*Janthinobacterium agaricidamnosum* [HG322949], *Collimonas pratensis* [CP013234] and *Herbaspirillum seropedicae* [CP011930]) were used as outgroups. Maximum likelihood (IQTREE) and Bayesian analyses (MrBayes) were performed based on a concatenated set of 108 proteins. Maximum likelihood tree is shown. SH-aLRT support (%) and ultrafast bootstrap support (%) values based on 1000 replicates, and Bayesian posterior probabilities are indicated on the internal nodes. Asterisks stand for a maximal support in each analysis (100% / 1).

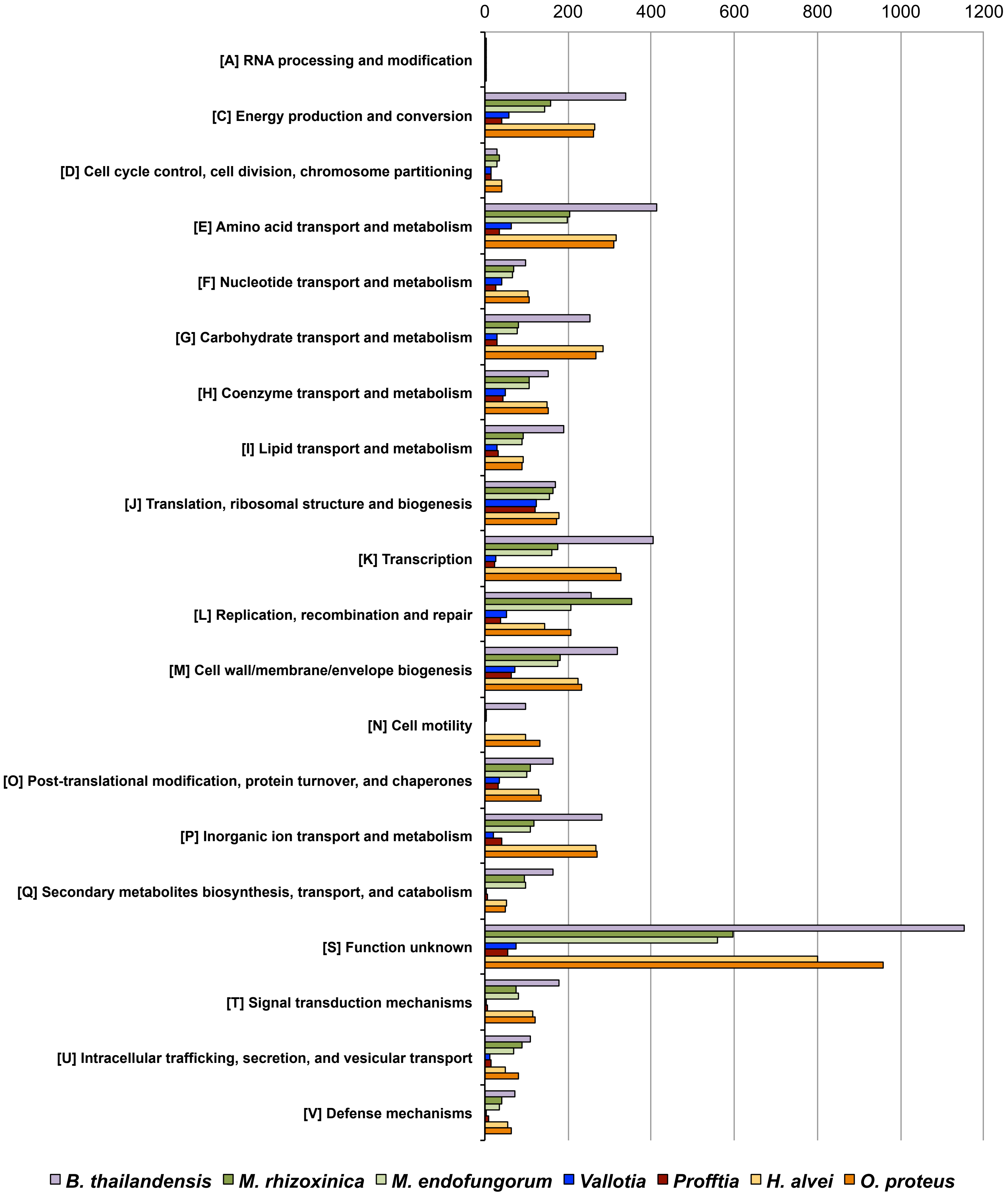

Figure S4. Functional reduction in *Vallotia* and *Profftia*, as shown by the number of genes among main functional categories according to the EggNOG classification in the genomes of *Profftia*, *Vallotia*, and closely related bacteria. The fungus-associated endosymbionts, *Mycetohabitans rhizoxinica* [FR687359.1, FR687360.1, FR687361.1], and *M. endofungorum* [GCA_002927045.1] are the closest relatives of *Vallotia*. Free-living bacteria involve the *Vallotia*-related *Burkholderia thailandensis* [CP008785.1], and *Profftia*-related *Hafnia alvei* [CP036514.1], and *Obesumbacterium proteus* [CP014608.1].

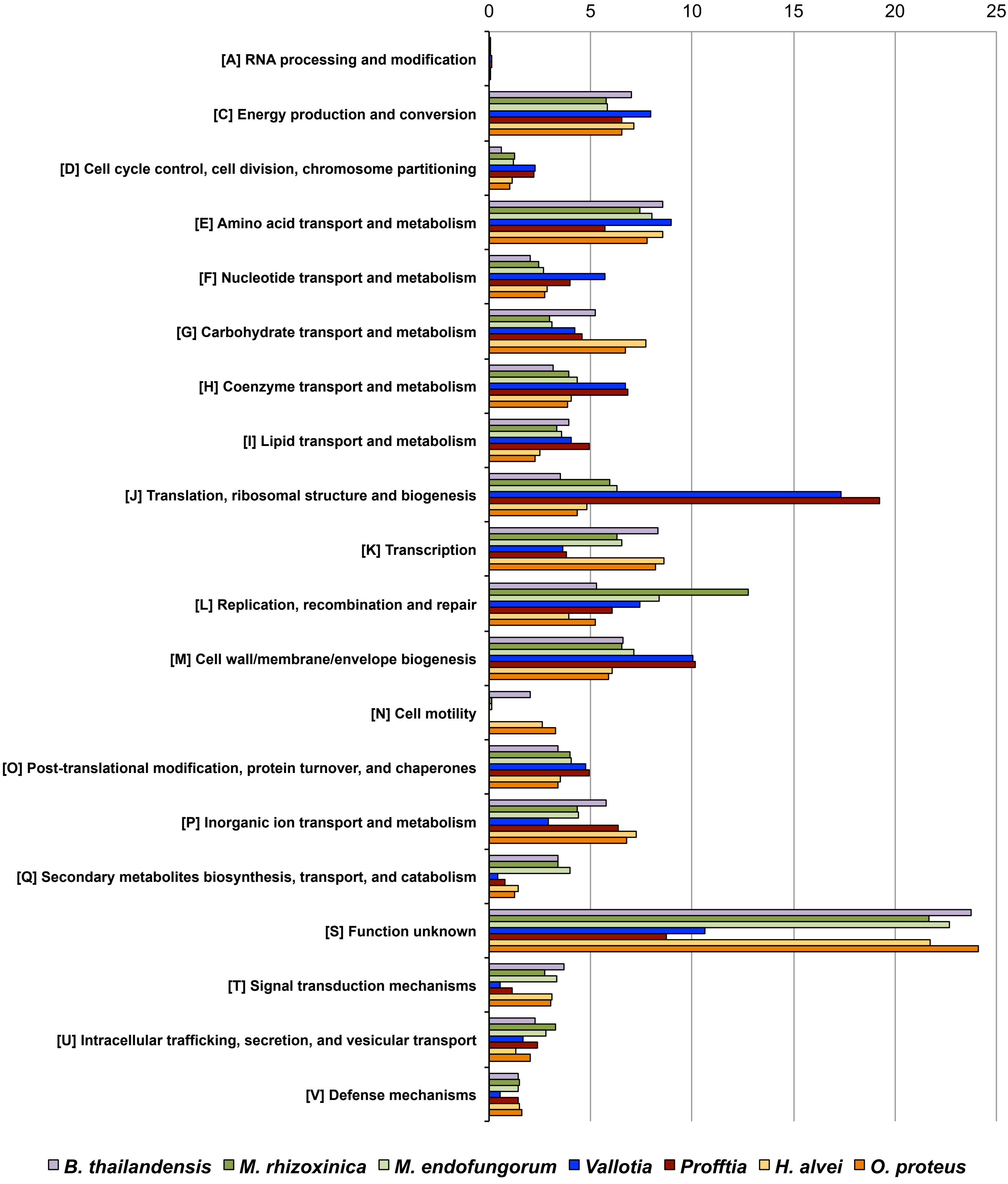

Figure S5. Proportion (%) of genes among main functional categories according to the EggNOG classification in the genomes of *Profftia*, *Vallotia*, and closely related bacteria. The fungus-associated endosymbionts, *Mycetohabitans rhizoxinica* [FR687359.1, FR687360.1, FR687361.1], and *M. endofungorum* [GCA_002927045.1] are the closest relatives of *Vallotia*. Free-living bacteria involve the *Vallotia*-related *Burkholderia thailandensis* [CP008785.1], and *Profftia*-related *Hafnia alvei* [CP036514.1], and *Obesumbacterium proteus* [CP014608.1].

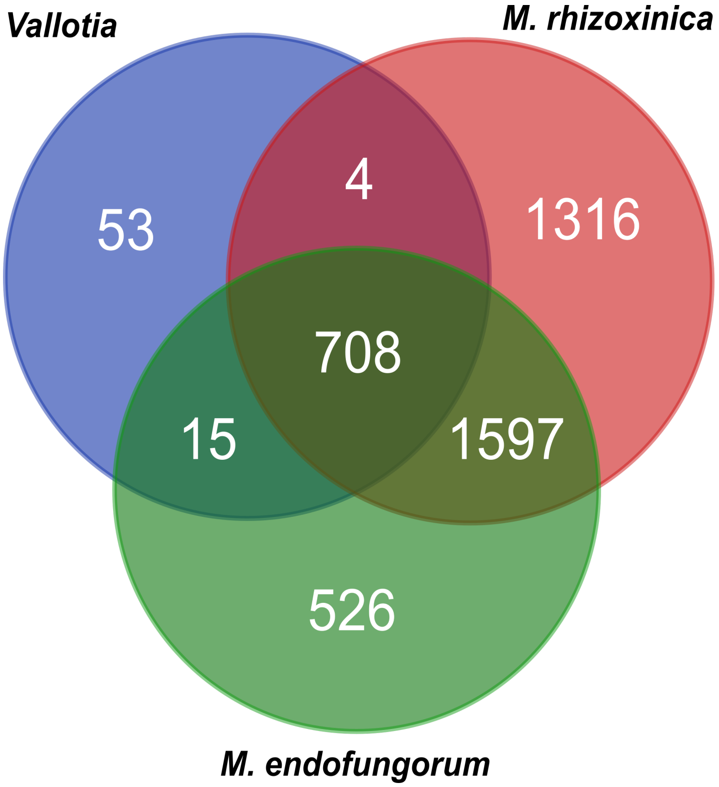

Figure S6. Venn diagram showing the pan-genome of the insect endosymbiont, *Vallotia*, and related fungus-associated endosymbionts, *M. rhizoxinica* [FR687359.1, FR687360.1, FR687361.1] and *M. endofungorum* [GCA_002927045.1].

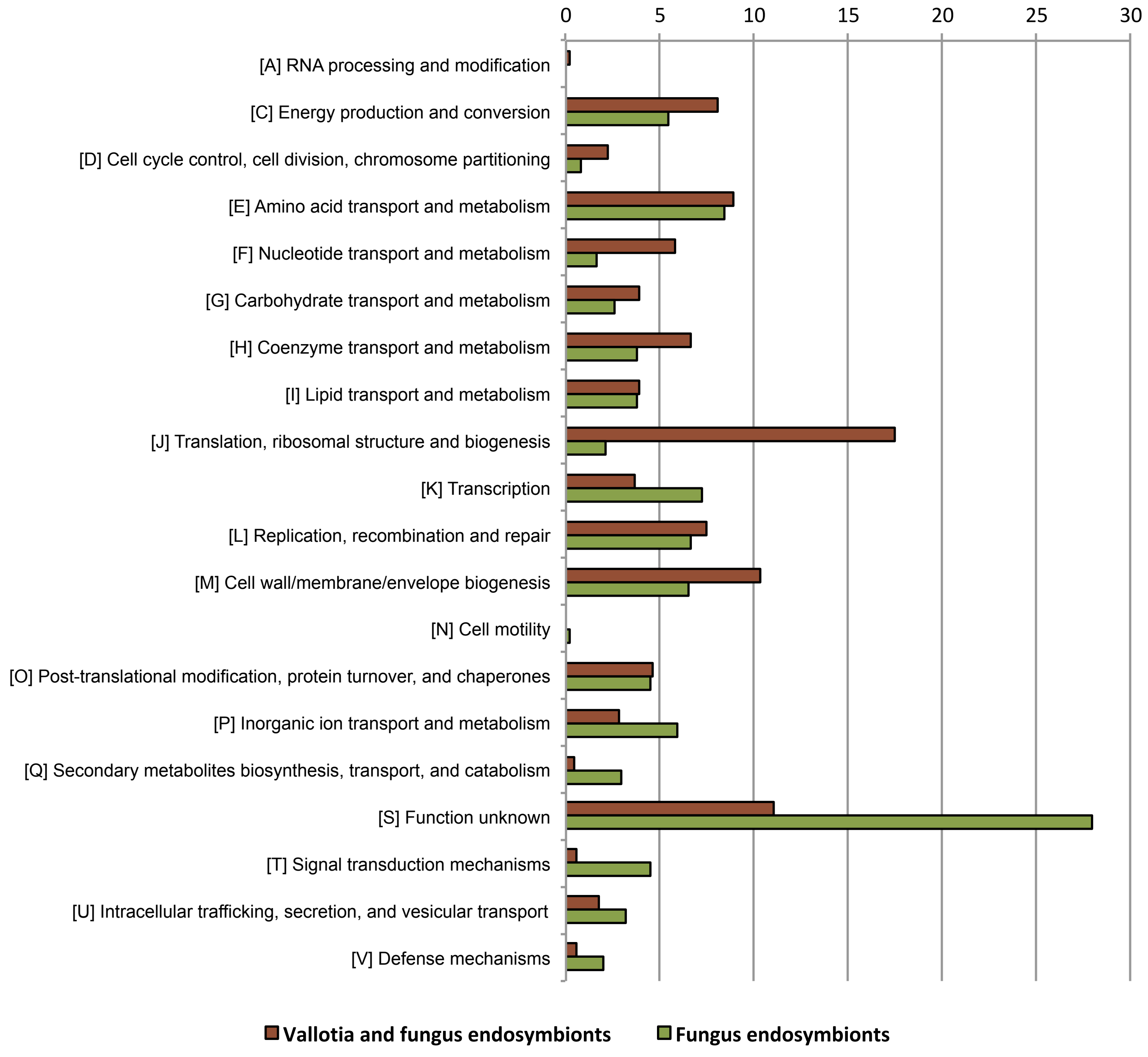

Figure S7. Proportion (%) of genes shared by *Vallotia* and fungus-associated endosymbionts (shown in brown) compared to those shared only by the fungus-associated endosymbionts (shown in green) – *M. rhizoxinica* [FR687359.1, FR687360.1, FR687361.1] and *M. endofungorum* [GCA_002927045.1] – among the main functional categories according to the EggNOG classification.

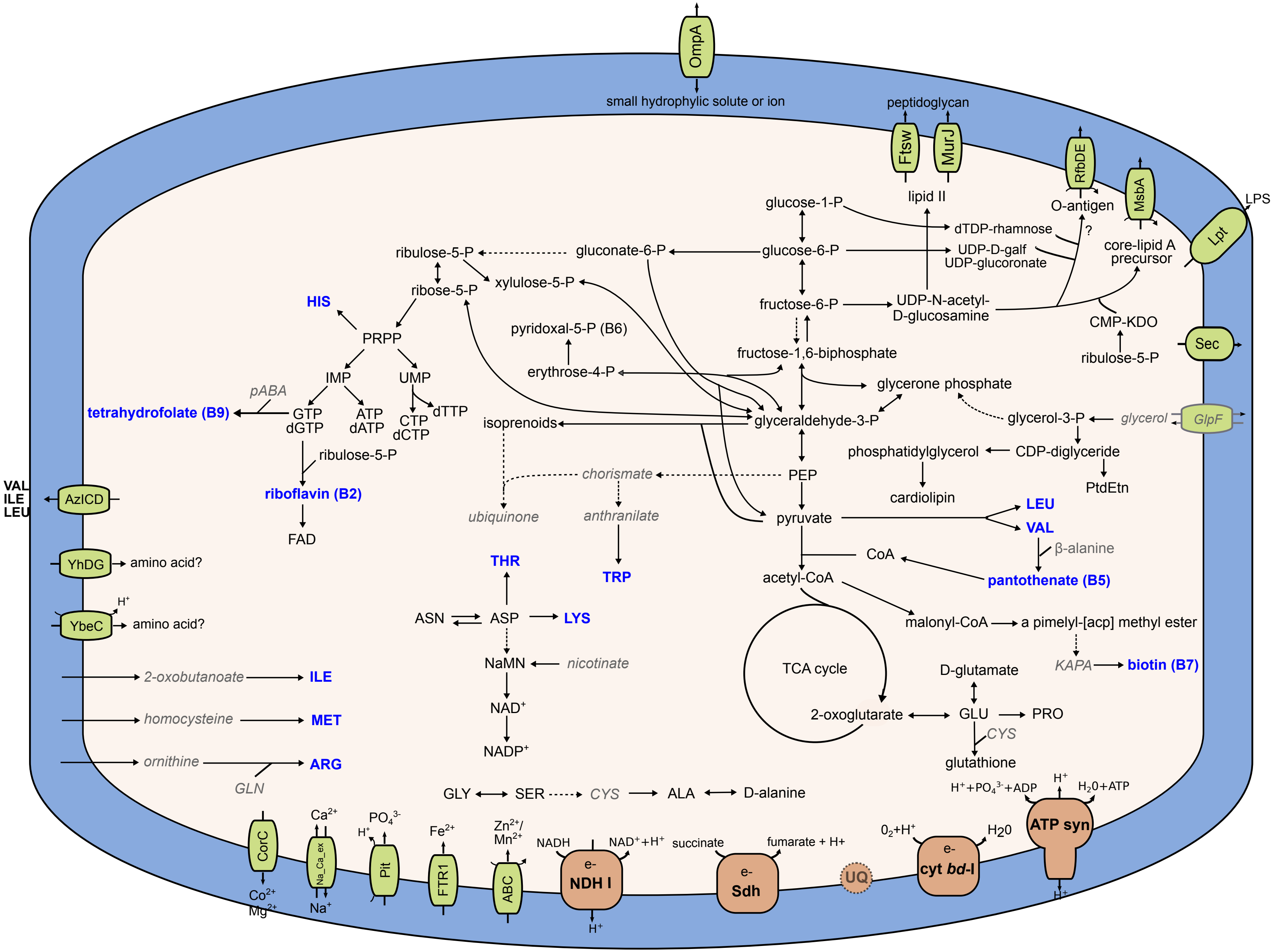

Figure S8. Metabolic pathway reconstruction of *Vallotia*. Dashed lines indicate missing or incomplete pathways. Compounds of which biosynthesis or transport could not be confirmed based on the genomic data are in grey italics. Essential amino acids and B vitamins produced by *Vallotia* are shown in blue.

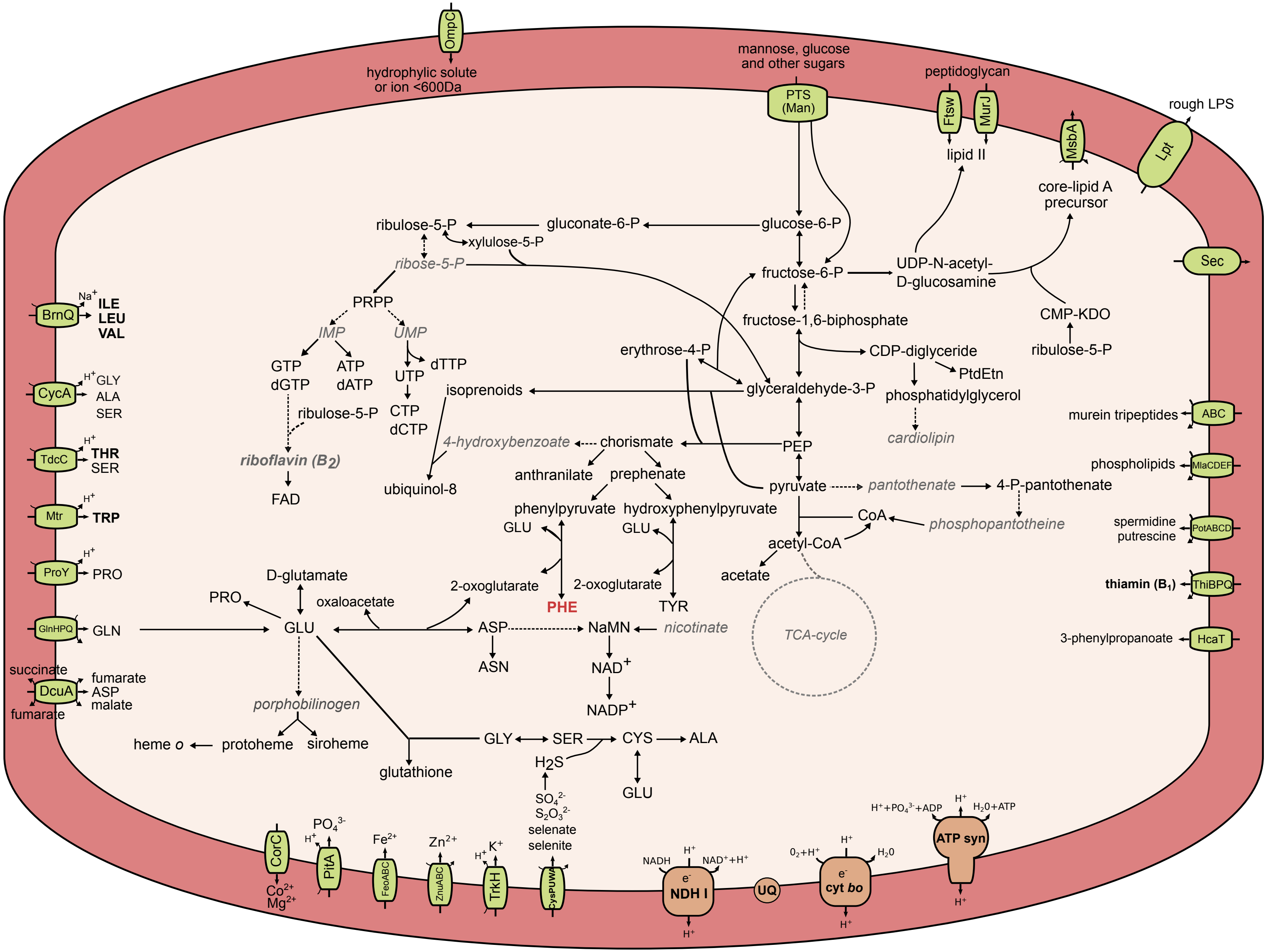

Figure S9. Metabolic pathway reconstruction of *Profftia*. Dashed lines indicate missing or incomplete pathways. Compounds of which biosynthesis or transport could not be confirmed based on the genomic data are in grey italics. Bolded texts indicate essential amino acids and B vitamins. Essential amino acids and B vitamins produced by *Profftia* are shown in red.

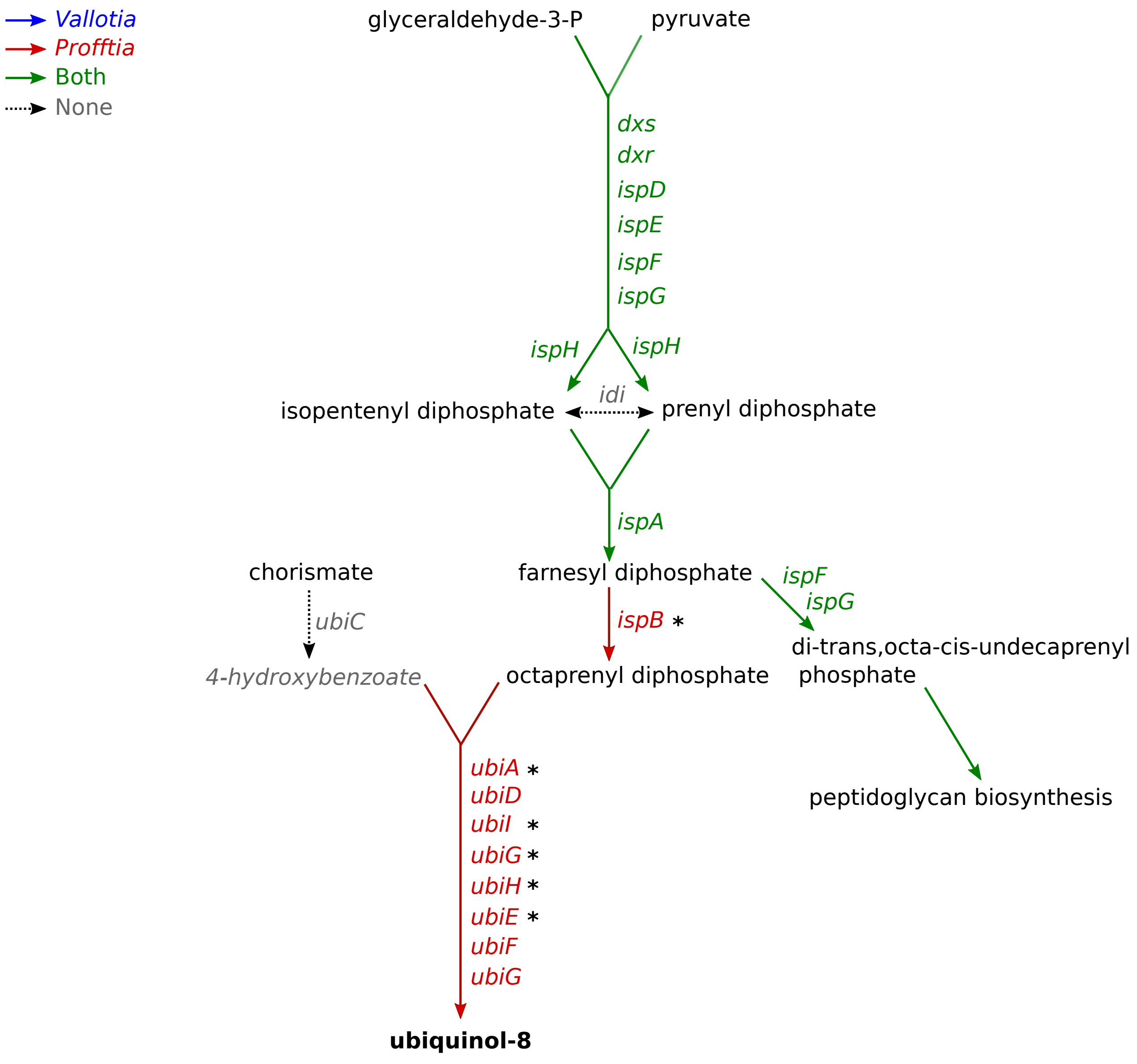

Figure S10. Ubiquinone biosynthesis in *Profftia* and *Vallotia*. *Profftia* has a complete gene set for the pathway. Asterisks indicate pseudogenized genes in *Vallotia*, which is not able to synthesize ubiquinone. Farnesyl diphosphate can be used for peptidoglycan synthesis by both symbionts.

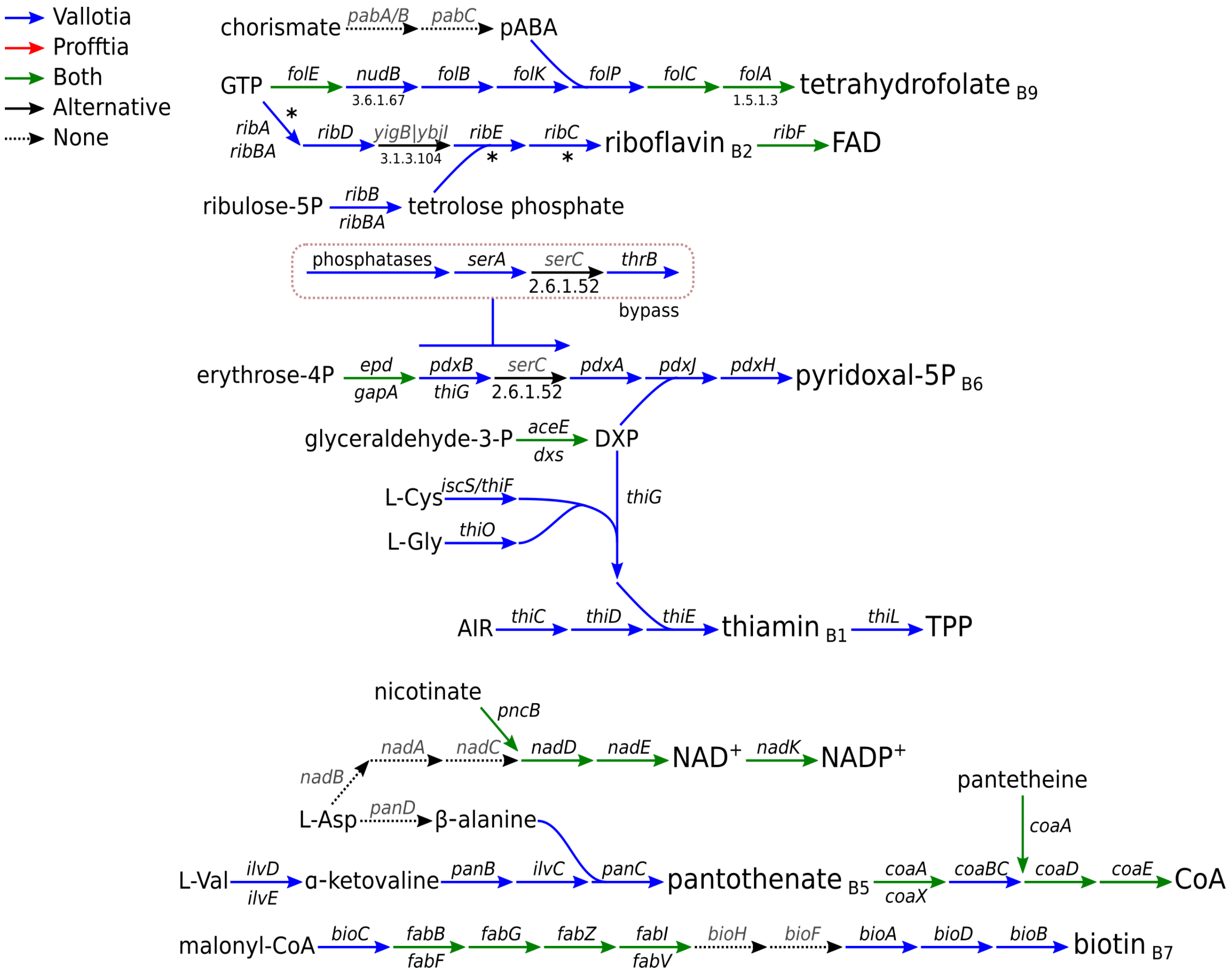

Figure S11. B vitamin synthesis as inferred based on the presence of genes in *Vallotia* and *Profftia*. Missing genes are shown in grey. Asterisks indicate pseudogenes of *Profftia*.

Supplementary references

1. Toenshoff, E.R., Gruber, D., and Horn, M. Co-evolution and symbiont replacement shaped the symbiosis between adelgids (Hemiptera: Adelgidae) and their bacterial symbionts. Environ Microbiol 2012; 14, 1284–1295.

2. von Dohlen, C.D., Spaulding, U., Patch, K.B., Weglarz, K.M., Foottit, R.G., Havill, N.P., *et al*. Dynamic acquisition and loss of dual-obligate symbionts in the plant-sap-feeding Adelgidae (Hemiptera: Sternorrhyncha: Aphidoidea). Front Microbiol 2017; 8:1037.

3. Haase, I., Sarge, S., Illarionov, B., Laudert, D., Hohmann, H.-P., Bacher, A., *et al.* Enzymes from the Haloacid Dehalogenase (HAD) superfamily catalyse the elusive dephosphorylation step of riboflavin biosynthesis. Chembiochem 2013; 14: 2272–2275.

4. Kuznetsova, E., Proudfoot, M., Gonzalez, C.F., Brown, G., Omelchenko, M. V., Borozan, I., *et al.* Genome-wide analysis of substrate specificities of the *Escherichia coli* haloacid dehalogenase-like phosphatase family. J Biol Chem 2006; 281: 36149–36161.

5. Kim, J., Flood, J.J., Kristofich, M.R., Gidfar, C., Morgenthaler, A.B., Fuhrer, T., *et al.* Hidden resources in the *Escherichia coli* genome restore PLP synthesis and robust growth after deletion of the essential gene *pdxB*. Proc Natl Acad Sci USA 2019; 116: 24164–24173.

6. Oberhardt, M.A., Zarecki, R., Reshef, L., Xia, F., Duran-Frigola, M., Schreiber, R., *et al.* Systems-wide prediction of enzyme promiscuity reveals a new underground alternative route for pyridoxal 5'-phosphate production in *E. coli*. PLoS Comput Biol 2016; 12: e1004705.

7. Manzano-Marín, A., Coeur d'acier, A., Clamens, A.-L., Orvain, C., Cruaud, C., Barbe, V., *et al.* E. A freeloader? The highly eroded yet large genome of the *Serratia symbiotica* symbiont of *Cinara strobi*. Genome Biol Evol 2018; 10: 2178–2189.

8. Manzano-Marín, A., Coeur d'acier, A., Clamens, A.-L., Orvain, C., Cruaud, C., Barbe, V., *et al.* E. Serial horizontal transfer of vitamin-biosynthetic genes enables the establishment of new nutritional symbionts in aphids' di-symbiotic systems. ISME J 2020; 14: 259–273.
